# Genome-Wide Identification and Expression Analysis of Thionin Family in Rice (*Oryza sativa*) and Functional Characterization of *OsTHION15* in Drought Stress and ABA Stress

**DOI:** 10.3390/ijms26073447

**Published:** 2025-04-07

**Authors:** Maokai Yan, Mengnan Chai, Chang An, Xiaohu Jiang, Fan Yang, Xunlian Fang, Tingyu Liu, Yunfei Ju, Boping Tang, Hanyang Cai, Yuan Qin

**Affiliations:** 1 College of Agriculture, Guangxi University, Nanning 530004, China; yanmaokai123@163.com; 2 Key Laboratory of Genetics, Breeding and Multiple Utilization of Crops, Ministry of Education, Fujian Provincial Key Laboratory of Haixia Applied Plant Systems Biology, College of Life Sciences, Fujian Agriculture and Forestry University, Fuzhou 350002, China; chaimengnan1@163.com (M.C.); ancher0928@163.com (C.A.);; 3 Jiangsu Provincial Key Laboratory of Coastal Wetland Bioresources and Environmental Protection, Jiangsu Collaborative Innovation Center for Coastal Biology and Agriculture, School of Wetlands, Yancheng Teachers University, Yancheng 224002, China

**Keywords:** rice, OsTHION family, gene expression patterns, *OsTHION15*, stress responses

## Abstract

The *OsTHION* family represents a class of cysteine-rich signal peptides widely recognized for their significant roles in plant disease resistance and immunity. While members of this family are known to be induced under various biotic and abiotic stresses, their responses to environmental stressors beyond disease resistance remain underexplored. This study investigates the evolution, expression patterns, and functional roles of the *OsTHION* gene family in rice (*Oryza sativa*) under diverse stress conditions. Using sequence data from the Phytozome database, we identified 44 *OsTHION* family members and classified them into four groups based on phylogenetic analysis. *Cis*-acting element analysis revealed that the promoter regions of *OsTHION* genes are enriched with regulatory elements associated with light response, hormone signaling, plant growth, and stress responses. The *OsTHION* genes exhibit complex organ-specific expression patterns, with *OsTHION30* and *OsTHION36* showing ubiquitous expression, while other members are highly expressed in specific tissues or developmental stages. Under drought, salt, and low-temperature stress, *OsTHION* genes undergo significant expression changes, underscoring their critical role in plant adaptation to environmental challenges. Notably, *OsTHION15* was markedly upregulated under drought stress, and the *Osthion15* mutant displayed heightened sensitivity to drought and ABA stress, confirming its pivotal role in stress resistance. RNA sequencing analysis identified many differentially expressed genes (DEGs), primarily enriched in pathways related to ribosomal function and plant hormone signaling, suggesting that *OsTHION15* may regulate stress responses through multiple mechanisms. In summary, this study advances our understanding of the *OsTHION* gene family and highlights its intricate involvement in regulating rice growth, development, and environmental stress responses. These findings offer valuable insights and technical support for crop improvement, with potential applications in enhancing environmental adaptability and yield stability in crops.

## 1. Introduction

In the natural environment, plants are constantly exposed to a variety of environmental stresses. Over evolutionary time, they have developed sophisticated and efficient defense mechanisms to ensure growth and survival. These mechanisms include physical and chemical defenses [1,2], as well as the innate immune system. When activated, the innate immune system triggers defense-related signaling pathways and promotes the accumulation of antimicrobial proteins, such as pathogenesis-related (PR) proteins, to counteract pathogen invasion [3]. PR proteins are typically small molecules with molecular weights ranging from 5 to 75 kDa [4]. Among them, plant thionins represent a crucial class of antimicrobial peptides and are key members of the PR protein family, playing a vital role in plant defense mechanisms [4]. These peptides are widely distributed across the plant kingdom and are known for their potent bactericidal effects, achieved by disrupting bacterial, fungal, and other cellular membranes. As a result, thionins are integral to the innate immune response of plants [5]. Thionins are alkaline, sulfur-rich peptides due to their high cysteine content, with a molecular mass typically between 5 and 10 kDa and a sequence length of approximately 45 to 90 amino acids. Structurally, they consist of three main regions: an N-terminal signal peptide, a central thionin domain, and a C-terminal acidic region. Upon maturation, they form disulfide bridges from 6 to 14 cysteine residues, which confer structural stability and functional activity [6,7,8].

Current research indicates that plant thionins primarily interact with negatively charged phospholipids in bacterial cell membranes. This interaction disrupts membrane integrity, leading to an imbalance of intracellular ions such as Ca^2^⁺ and K⁺. Consequently, essential cellular components, including proteins and nucleotides, leak out, exerting toxic effects on bacteria and protecting plants from bacterial invasion [9,10,11,12]. For example, the defense factor CpTHION II, purified from rainbow beans, effectively inhibits *Fusarium oxysporum* activity at 100 μg/mL concentration by altering fungal hyphal membrane permeability, thereby protecting wheat grains from fungal damage [13]. In chili pepper (*Capsicum annuum*), the thionin protein CaTHION enhances resistance to bacterial pathogens by inducing the endogenous production of hydrogen peroxide (H₂O₂) within *Fusarium oxysporum* f. sp. *papryticum* [14]. Overexpression of thionin genes has been shown to significantly enhance plant resistance to various pathogens. In rice, overexpression of *OsTHION1* increases resistance to necrotrophic bacteria [15]. Similarly, in citrus, overexpression of *CsTHIONF*, which encodes a sulfur-rich protein, reduces citrus canker symptoms and inhibits bacterial proliferation in transgenic plants [16]. In *Arabidopsis*, the expression of *AtTHION2*.1 is upregulated in response to fungal infection, and its overexpression significantly reduces chlorophyll degradation and fungal proliferation in *Arabidopsis* cotyledons [17,18]. The transfer of the *THION* gene from mistletoe into *Arabidopsis* enhances its defense against pathogenic attacks [19]. In chili peppers (*Capsicum annuum*), anthracnose infection triggers the rapid induction of the *PeTHION* gene within the fruit, protecting against fungal invasion and safeguarding plant tissues and organs [20]. Beyond bacterial and fungal resistance, thionins also exhibit insect-resistant properties. For instance, in barley, the expression of the *THION* genes is significantly upregulated under aphid stress, and its overexpression in tobacco reduces susceptibility to peach aphids [21,22]. In rice, the thionin gene *OsTHION15* inhibits Aspergillus oryzae activity and *Fusarium oxysporum* hyphal growth, and its transfer into tobacco enhances antibacterial properties [23]. Additionally, overexpression of *OsTHION7* in rice significantly reduces root sensitivity to pathogens [24]. These findings collectively underscore the critical role of thionins in enhancing plant innate immunity and highlight their potential for developing disease-resistant crops through genetic engineering.

Studies have demonstrated that pathogenesis-related proteins (PRs) confer disease resistance and are induced by abiotic stress factors such as cold damage, drought, heavy metals, and mechanical injury [4]. For example, the PR-10 protein is induced by salt and heavy metal stress, enhancing salt and drought tolerance in rice and potatoes [25,26,27]. In *Arabidopsis*, salt and drought stress significantly increase the expression of PR genes [28,29]. In pepper plants, the mRNA levels of PR1, a marker gene for systemic acquired resistance, rise in response to abiotic stress [30]. Classic antifungal PR proteins, such as PR2 and PR3, protect against cell damage caused by cold stress and exhibit antifreeze activity [31]. Additionally, cold stress significantly induces the expression of antimicrobial peptides (AMPs), specifically PR12 and PR13 (THION), in oxytropis and wheat plants [32,33]. However, research on Cys-rich THION peptides has primarily focused on their role in disease resistance, with limited exploration of their responses to abiotic stress and hormone signaling. Previous studies have shown that *OsTHION* genes in rice are induced by various biotic and abiotic stresses [34], suggesting their potential importance in abiotic stress responses.

In this study, we comprehensively analyzed OsTHION proteins in rice, including phylogenetic tree construction, prediction of *cis*-acting regulatory elements, and profiling of tissue-specific and stress-responsive expression patterns. We performed a detailed functional analysis of *OsTHION15* to elucidate its role and response mechanisms under ABA treatment. Using RNA-seq transcriptomic analysis, we characterized the altered gene expression profiles in the *Osthion15* mutant under ABA and drought stress. Our findings provide significant insights into the molecular mechanisms by which *OsTHION15* regulates plant responses to ABA and drought stress. These results contribute to a broader understanding of rice stress tolerance and offer potential genetic improvement targets to enhance crop resilience under adverse environmental conditions.

## 2. Results

### 2.1. Evolutionary Analysis of OsTHION Genes

**Figure 1:**
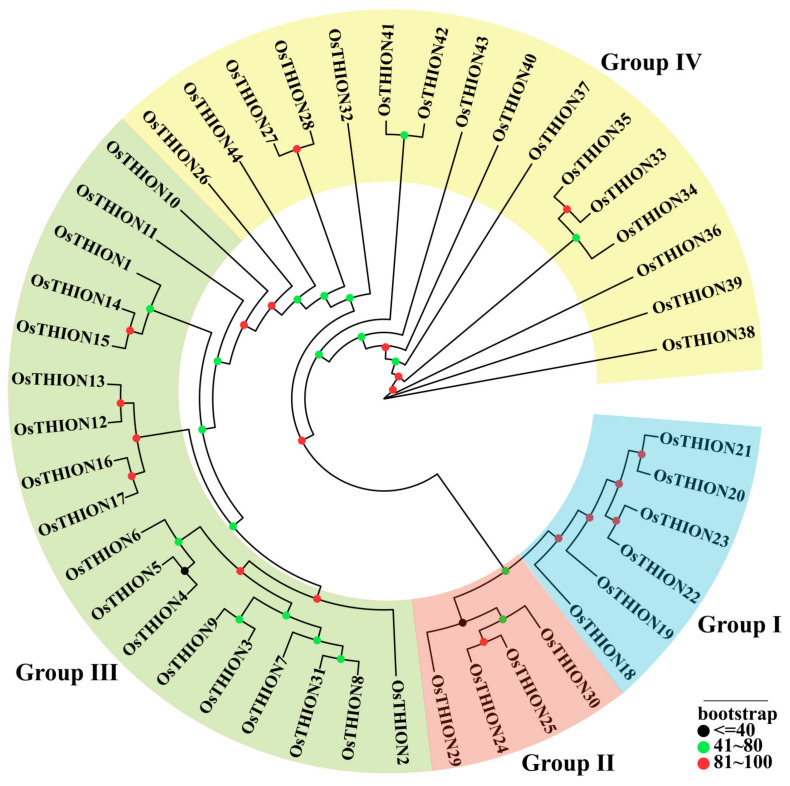
Phylogenetic tree analysis of the coding sequences of *OsTHION* family genes of rice. All 4 groups of OsTHIONs were well separated in different clades and represented by different colors.

The sequence information for 44 *OsTHION* family genes, including amino acid and promoter sequences, was obtained from the Phytozome database (https://phytozome-next.jgi.doe.gov/) (accessed on 12 August 2024) (Table S1). To investigate the phylogenetic relationships among OsTHION family members, a maximum likelihood phylogenetic tree was constructed using MEGA software (Figure 1). Based on the tree’s topological structure, the OsTHION proteins were classified into four groups (I–IV). Group III contained the highest number of members (*n* = 18), followed by Group IV (*n* = 16) and Group I (*n* = 9), while Group II was the smallest, with only four members. To further explore the evolutionary relationships of OsTHIONs, 62 AtTHION family members were identified through BLAST (version2.16.0) analysis using the 44 OsTHION protein sequences (Table S2).

A phylogenetic tree incorporating both the OsTHION and AtTHION proteins revealed that the four OsTHION subgroups clustered separately, indicating a distant evolutionary relationship with AtTHION proteins. Notably, *OsTHION31* is the only member that showed a close relationship with *AT1G53282* and *AT1G53285* (Figure S1). Collinearity analysis further confirmed the lack of synteny between OsTHIONs and AtTHIONs (Figure S2), suggesting that the OsTHION family has undergone significant functional divergence between monocotyledonous and dicotyledonous plants.

### 2.2. Cis-Acting Element Analysis of OsTHION Genes

**Figure 2:**
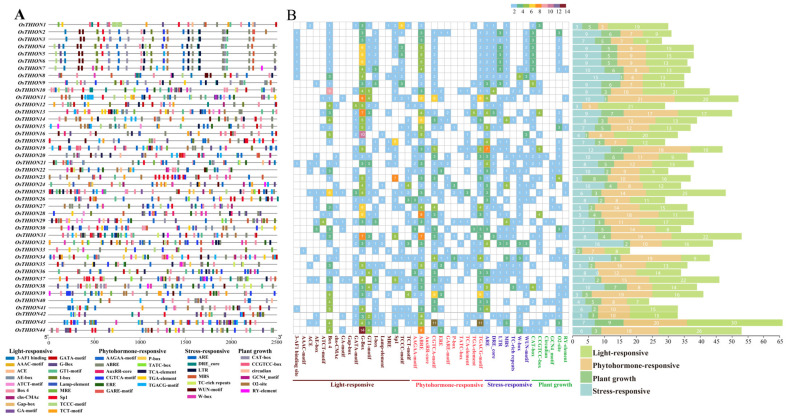
Regulatory elements in the promoter region of *OsTHION* genes in rice. (**A**) Distribution of cis-acting elements identified in the 2500 bp upstream region of *OsTHION* genes. Different colors represent the different types of cis-elements. (**B**) The number of cis-acting elements on putative promoters of *OsTHION* genes.

To identify potential regulatory mechanisms, 2500 bp upstream promoter sequences of the 44 *OsTHION* genes were analyzed for *cis*-acting elements. A total of 42 different types of cis-acting elements were identified, including 18 light-responsive elements, 11 hormone-related elements, 7 stress-responsive elements, and 10 elements related to plant growth and development, with respective counts of 639, 498, 326, and 160 across the promoters of the 44 *OsTHION* genes (Figure 2A). Among these elements, those involved in light responses were the most abundant. Notably, G-box elements, which play an important role in regulating plant light-induced gene expression and mediating the signal transduction of photosensitive pigments [23,35], were particularly prevalent in the promoters of the OsTHION family genes. For instance, *OsTHION16* and *OsTHION44* promoters contained 10 and 14 G-box elements, respectively (Figure 2B), suggesting their potential roles in light-induced gene expression. Additionally, abscisic acid-responsive elements (ABRE) and jasmonic acid-responsive elements (CGTCA) were also highly represented, with ABRE elements identified in 43 *OsTHION* promoters and CGTCA elements in 38 (Figure 2B). These results indicate that *OsTHION* genes are likely involved in the ABA and JA signaling pathways, which are critical for stress responses.

### 2.3. The Expression Pattern of OsTHION Family Genes in Rice Tissues

**Figure 3:**
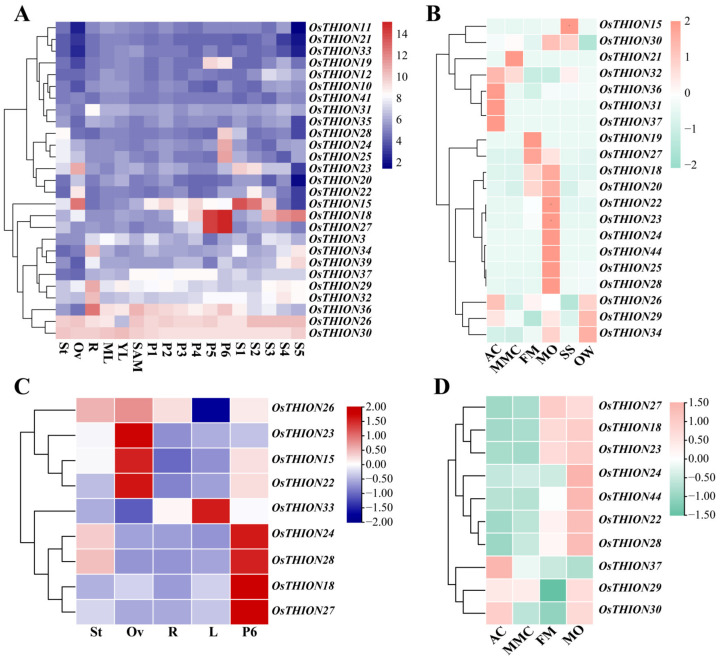
The expression profiles of selected *OsTHION* genes. (**A**) Expression profiles for mature stigma (St) and ovary (Ov), seedling root (R), mature (ML) and young leaf (YL), and shoot apical meristem (SAM), as well as for panicle (P1 to P6) and seed (S1 to S5) developmental stages, were obtained from the rice eFP browser. (**B**) Expression profiles for four stages of the ovule (AC, MMC, FM, and MO), stigma and style (SS), and the ovary wall (OW) of the FM stage. (**C**) qRT-PCR results for mature stigma (St) and ovary (Ov), seedling root (R), and young leaf (YL). (**D**) qRT-PCR results for four stages of the ovule (AC, MMC, FM, and MO). The expression heatmap (**A**,**B**) is based on the FPKM values retrieved from the browser. Pixel intensities are proportional to the actual expression levels, which were calculated as log2 expression values. The heatmap of qRT-PCR (**C**,**D**) represents the average relative expression levels calculated based on CT values and a normalized presentation of the relative expression levels. Three biological replicates, each with three technical repeats, were conducted for each sample. Data are mean values ± SDs.

To investigate the potential functions of *OsTHIONs*, the expression levels (FPKM) of 27 *OsTHION* genes across different tissues were analyzed using the Rice eFP Browser [36] (Table S3). A heatmap was constructed to visualize the expression patterns of these 27 genes in the stigma and ovary of mature inflorescences, as well as in the roots and leaves of the 2-week-old seedlings and the panicles and seeds at different developmental stages (Figure 3A). The heatmap revealed that most *OsTHION* genes exhibit organ-specific expression patterns, except for *OsTHION30* and *OsTHION36*, which were ubiquitously expressed across all tissues (Figure 3A). Specifically, *OsTHION30* and *OsTHION36* showed high expression levels in all vegetative tissues and at multiple floral development stages, suggesting their broad roles in rice growth and development. In contrast, *OsTHION18*, *OsTHION24*, *OsTHION25*, *OsTHION27*, and *OsTHION28* were specifically expressed in the late-stage panicles, indicating their potential involvement in the later stages of flower development (Figure 3A). Similarly, *OsTHION29*, *OsTHION31*, *OsTHION32*, and *OsTHION34* were predominantly expressed in the seedling roots (Figure 3A), implying their functional importance in root development. Additionally, *OsTHION22* and *OsTHION23* were explicitly expressed in the mature ovaries (Figure 3A), highlighting their potential roles in the late stages of ovary development. To validate these expression patterns, qRT-PCR analysis was performed on nine *OsTHION* genes in the roots and leaves of the seedlings, as well as in the stigmas and ovaries of the mature panicles. The qRT-PCR results were consistent with the FPKM heatmap data, confirming the tissue-specific expression patterns of these genes (Figure 3C).

In addition, an expression heatmap of 20 *OsTHION* genes was constructed based on FPKM expression data from female reproductive tissues of rice, including ovules at four developmental stages (AC, archesporial cell; MMC, megaspore mother cell; FM, functional megaspore; and MO, mature ovule), as well as the ovary wall (OW) and the style and stigma (SS) from FM-stage spikelets [37]. The heatmap revealed that *OsTHION* genes exhibit stage-specific expression patterns during ovule development. For instance, *OsTHION31*, *OsTHION36*, and *OsTHION37* were predominantly expressed during the AC stage, while *OsTHION21* showed specific expression during the MMC stage (Figure 3B), suggesting their critical roles in early ovule development, including female germ cell fate determinations and megaspore mother cell formations. Furthermore, *OsTHION19* and *OsTHION27* were highly expressed during the FM stage, while six *OsTHION* genes (*OsTHION22*, *OsTHION23*, *OsTHION24*, *OsTHION25*, *OsTHION28*, and *OsTHION44*) were specifically expressed at the mature ovule (MO) stage (Figure 3B). These stage-specific expression patterns indicate their involvement in late ovule development or fertilization regulation in rice. To further validate these findings, qRT-PCR analysis of 10 *OsTHION* genes was conducted to validate their expression patterns across the four ovule developmental stages. The qRT-PCR results confirmed the stage-specific expression patterns observed in the heatmap, further supporting the functional importance of *OsTHION* genes during ovule development (Figure 3D).

### 2.4. Expression Analysis of OsTHIONs Under Stress Conditions

**Figure 4:**
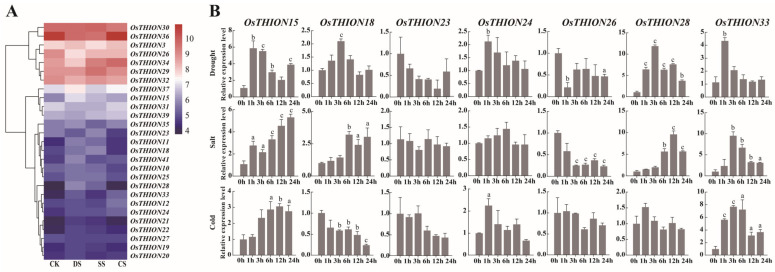
The expression profiles of selected *OsTHION* genes under abiotic stress. (**A**) The expression heatmap of *OsTHION* genes. (**B**) The qRT-PCR results of selected *OsTHION* genes. Three biological replicates, each with three technical repeats, were conducted for each sample. Data are mean values ± SDs. a, *p* < 0.05, b, *p* < 0.01, c, *p* < 0.001 by one-way ANOVA.

Analysis of *cis*-acting elements within the promoter regions of *OsTHION* genes revealed an abundance of stress-responsive elements, suggesting a significant role for *OsTHIONs* in rice stress responses. To further elucidate their functions under stress conditions, expression heatmaps were generated using FPKM values from 27 *OsTHION* genes subjected to drought, salt, and cold stress (Table S4). The results demonstrated substantial variation in *OsTHION* expression levels across these treatments (Figure 4A). Under drought stress, the expression levels of 12 *OsTHION* genes were significantly upregulated, with *OsTHION28*, *OsTHION15*, *OsTHION11*, and *OsTHION44* showing the most pronounced increases. Similarly, 11 *OsTHION* genes exhibited elevated expression under salt stress, with OsTHION28 displaying the highest upregulation. In response to cold stress, nine *OsTHION* genes were upregulated, with *OsTHION33* and *OsTHION34* showing the most significant induction. These findings indicate that *OsTHION* genes actively respond to drought, salt, and cold stress. Notably, *OsTHION15*, *OsTHION24*, and *OsTHION33* were significantly upregulated under all three stress conditions (Figure 4A), suggesting their broad roles in plant stress adaptation. Furthermore, the *OsTHION* genes exhibited stress-specific expression patterns. For example, *OsTHION19* and *OsTHION41* were exclusively induced under drought stress, while *OsTHION34* and *OsTHION29* were uniquely upregulated under salt stress. In contrast, *OsTHION46* was specifically induced only by cold stress (Figure 4A). These distinct expression profiles highlight the functional diversity of *OsTHION* genes in mediating stress-specific responses. To validate these findings, qRT-PCR experiments were conducted on selected *OsTHION* genes under drought, salt, and cold conditions (Table S5). The qRT-PCR results were consistent with the heatmap data. Specifically, *OsTHION15* and *OsTHION33* showed significant upregulation under all three stress conditions, while *OsTHION18* and *OsTHION28* were significantly upregulated under both drought and salt stress (Figure 4B). Notably, *OsTHION26* was consistently downregulated across all stress treatments, as observed in both the heatmap and qRT-PCR analyses (Figure 4B). These results suggest that *OsTHION26* may act as a negative regulator of stress responses, further underscoring the complexity of the *OsTHION* regulatory network in rice stress adaptation.

### 2.5. The Expression Pattern and Subcellular Localization

**Figure 5:**
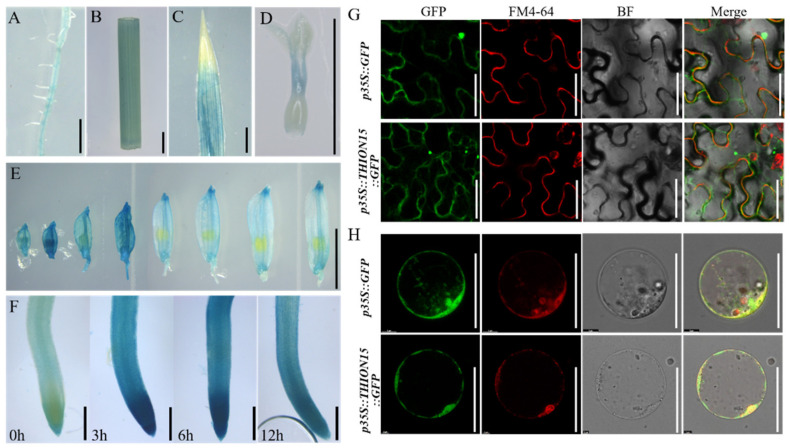
Expression pattern of *OsTHION15* and subcellular localization of the encoded protein. (**A**–**F**) Expression pattern of *pOsTHION15::GUS* in different rice tissues: (**A**) root, (**B**) stem, (**C**) leaf, (**D**) pistil at the FM stage, (**E**) florets at eight different developmental stages, and (**F**) seeding roots at different times under 150 mM mannitol treatment. (**G**,**H**) Subcellular localization of *p35S::OsTHION15::GFP* through transient expression in tobacco (**G**) and rice protoplasts (**H**). The upper row is the expression of *p35S::GFP* as the control. The GFP fluorescence channel is represented in green, and the FM4-64 fluorescence channel is shown in red. (**A**–**E**) Bar = 5 mm. (**F**) Bar = 1 mm. (**G**) Bar = 50 μm.

The expression level of *OsTHION15* was significantly elevated in response to drought, salt, and cold stress (Figure 4A,B), suggesting its crucial role in the stress response mechanisms of rice. To validate this hypothesis, *OsTHION15* was selected for further functional analysis. GUS staining of *pOsTHION15::GUS* transgenic plants revealed weak GUS signals in the roots, stems, and leaves of the seedlings (Figure 5A–C). Additionally, *OsTHION15* was expressed in the florets at various developmental stages (Figure 5E), with specific localization in the stigma and style (SS) of the pistil at the FM stage (Figure 5D). These findings suggest a potential role for *OsTHION15* in rice reproductive development, including flower organ formation and stamen–pistil interactions. To assess the response of *OsTHION15* to drought stress, *pOsTHION15::GUS* transgenic seedlings were subjected to 150 mM mannitol treatment as a drought mimic. GUS staining of the root samples at different time points indicated a significant increase in GUS signals after 3 h of mannitol exposure (Figure 5F), consistent with the expression heatmap and qRT-PCR data (Figure 4A,B). This confirms that drought stress induces the upregulation of *OsTHION15*. Subcellular localization using *p35S::OsTHION15::GFP* in *Nicotiana benthamiana* and rice protoplasts showed colocalization with the plasma membrane marker FM4-64 (Figure 5G,H), indicating that *OsTHION15* is localized to the plasma membrane. These findings suggest that *OsTHION15* functions in stress signaling and reproductive development, potentially through membrane-associated mechanisms (Figure 5G,H).

### 2.6. The Osthion15 Mutant Exhibits Increased Sensitivity to Drought Stress

**Figure 6:**
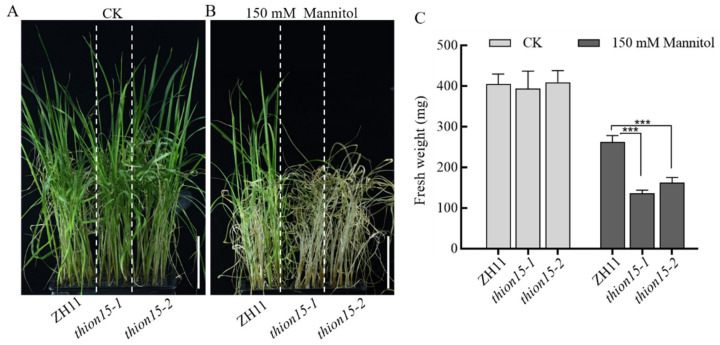
The phenotype of the *Osthion15* mutant under drought stress. (**A**) ZH11 and *Osthion15* grown in normal conditions as a control. (**B**) ZH11 and *Osthion15* grown under 150 mM mannitol for 10 days. (**C**) The quantification of fresh weight with a single plant. Data are mean values ± SDs. ***, *p* < 0.001 by one-way ANOVA. (**A**,**B**) Bar = 5 cm.

To elucidate the role of *OsTHION15* in drought stress response, we generated two homozygous *Osthion15* mutant lines, i.e., *Osthion15-1* and *Osthion15-2*, using gene editing technology. *Osthion15-1* carries a two-base (TT) deletion, while *Osthion15-2* has a single T-base deletion, both resulting in premature termination of protein translation (Figure S3). Two-week-old seedlings of the wild-type ZH11 and *Osthion15* mutants, grown in complete rice nutrient solution, were subjected to 150 mM mannitol treatment to simulate drought stress. Under normal conditions, the *Osthion15* mutants exhibited comparable seedling height and leaf color to ZH11 (Figure 6A). However, after 10 days of mannitol treatment, the *Osthion15* mutants displayed more severe wilting and higher mortality than ZH11 (Figure 6B). Fresh weight measurements further confirmed their increased drought sensitivity, with *Osthion15-1* and *Osthion15-2* showing significantly lower fresh weights (132 g and 162 g, respectively) compared to ZH11 (262 g) (Figure 6C). These results demonstrate that *Osthion15* mutants are more sensitive to drought stress, indicating that *OsTHION15* is critical in enhancing drought tolerance in rice.

### 2.7. The Osthion15 Mutant Exhibits Increased Sensitivity to ABA Stress

**Figure 7:**
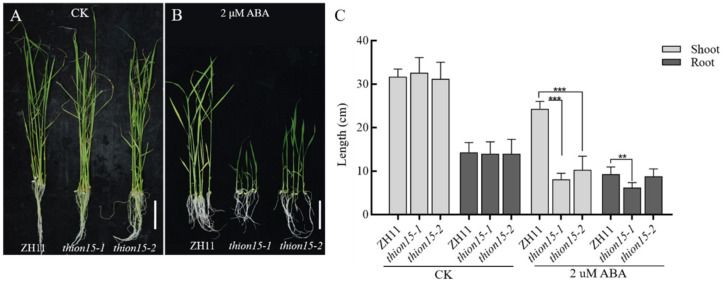
The phenotype of the *Osthion15* mutant under ABA stress. (**A**) ZH11 and *Osthion15* grown under 1/2 MS medium as a control. (**B**) ZH11 and *Osthion15* continuously grown under 1/2 MS medium containing 2 μM ABA for 20 days. (**C**) The quantification of the length of the shoots and roots. Data are mean values ± SDs. **, 0.001 < *p* < 0.01, ***, *p* < 0.001 by one-way ANOVA. (**A**,**B**) Bar = 5 cm.

The drought stress response in plants is closely associated with the abscisic acid (ABA) signaling pathway [38]. Notably, the *OsTHION15* promoter contains ABA-responsive cis-acting elements, such as ABREs, suggesting a potential role in ABA signaling. To explore this further, we examined the response of the *Osthion15* mutants to ABA stress. Seeds of ZH11 and the *Osthion15* mutants were germinated on standard 1/2 MS medium and 1/2 MS medium supplemented with 2 μM ABA. After 20 days, phenotypic observations and statistical analyses were conducted. Under normal conditions, the *Osthion15* mutants exhibited growth comparable to ZH11 (Figure 7A). However, under ABA stress, the *Osthion15* mutants exhibited significantly stunted growth compared to ZH11 (Figure 7B). Statistical analysis confirmed that the *Osthion15* mutants displayed pronounced growth defects under ABA stress, with *Osthion15-1* showing a more severe phenotype, likely due to premature protein truncation (Figure S3; Figure 7C). These findings indicate that the loss of *OsTHION15* function enhances ABA sensitivity, highlighting the involvement of *OsTHION15* in the ABA-mediated stress response pathway.

### 2.8. Differential Gene Expression Analysis of Osthion15 and ZH11 Under Drought and ABA Stress

**Figure 8:**
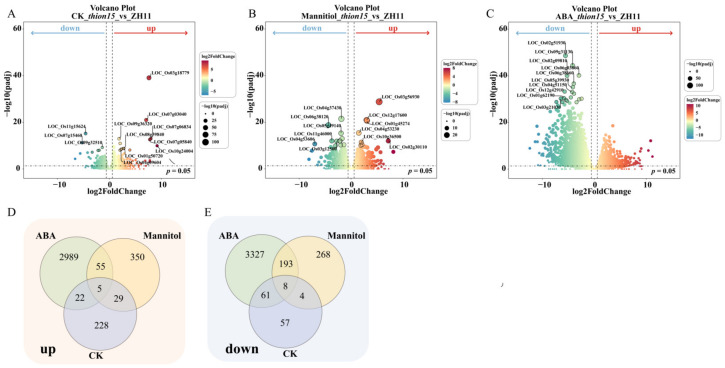
The RNA-seq analysis of the *Osthion15* mutant under CK, mannitol, and ABA stress. (**A**) Volcanic plot illustrating differentially expressed genes between the *Osthion15* mutant and ZH11 under CK conditions (**A**), mannitol (**B**), and ABA stress (**C**). (**D**,**E**). The Venn diagram illustrates the overlap in the number of genes that are concurrently upregulated (**D**) and downregulated (**E**) in both the *Osthion15* mutant and ZH11 under CK conditions, drought stress, and ABA stress.

To investigate the molecular mechanisms underlying *OsTHION15* in drought and ABA stress responses, RNA sequencing was performed on ZH11 and *Osthion15* seedlings under three conditions: normal growth (CK), mannitol-induced drought stress, and ABA treatment. Differentially expressed genes (DEGs) were identified using a fold change threshold of >2. Under CK conditions, *Osthion15* exhibited 414 DEGs compared to ZH11, with 284 upregulated and 130 downregulated genes (Figure 8A, Table S6). Under mannitol treatment, *Osthion15* showed 912 DEGs relative to ZH11, including 439 upregulated and 473 downregulated genes (Figure 8B, Table S6). ABA treatment induced the most significant transcriptional changes, with 6660 DEGs in *Osthion15* compared to ZH11, comprising 3071 upregulated and 3589 downregulated genes (Figure 8C, Table S6). Notably, *Osthion15* shared 60 upregulated and 201 downregulated genes under drought and ABA stress compared to ZH11 (Figure 8D,E, Table S6). Additionally, five upregulated and eight downregulated genes were consistently differentially expressed across the CK, drought, and ABA stress conditions (Figure 8D,E). These findings suggest that *OsTHION15* plays a central role in integrating drought and ABA stress responses at the transcriptional level.

To further explore the functional implications of these DEGs, Gene Ontology (GO) and Kyoto Encyclopedia of Genes and Genomes (KEGG) pathway analyses were conducted. Under CK conditions, no significant KEGG pathway enrichment was observed between *Osthion15* and ZH11. However, following ABA treatment, the DEGs were significantly enriched in pathways related to ribosomes, PPAR signaling, porphyrin metabolism, plant hormone signal transduction, MAPK signaling, and fatty acid degradation (Figure S4). Under drought stress, the DEGs were predominantly associated with biological processes, such as response to toxic substances, monovalent and inorganic ion transmembrane transport, and cellular-modified amino acid metabolism. The key enriched pathways included photosynthesis, nitrogen metabolism, and glyoxylate and dicarboxylate metabolism (Figure S4). These results highlight the distinct molecular pathways influenced by *OsTHION15* in response to drought and ABA stress, further supporting its regulatory role in stress adaptation.

### 2.9. Expression Profiles of Differentially Expressed Genes

**Figure 9:**
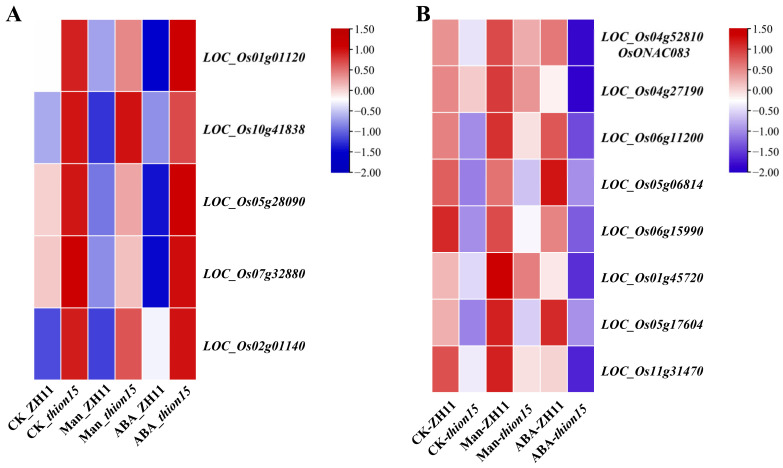
The expression heatmap of differential expression genes. (A-B) The expression heatmap of co-upregulated (**A**) and co-downregulated (**B**) genes under CK, drought, and ABA stress.

To explore the role of *OsTHION15* in rice drought and ABA responses, we analyzed and annotated differentially expressed genes (DEGs) in the *Osthion15* mutant compared to ZH11 under control (CK), drought, and ABA stress conditions. An expression heatmap based on FPKM values revealed distinct transcriptional changes. Five genes, *LOC_Os01g01120* (*OsEP1*), *LOC_Os10g41838* (*OsFIP*), *LOC_Os05g28090*, *LOC_Os07g32880*, and *LOC_Os02g01140* (*OsGDSL*), were consistently upregulated in the *Osthion15* mutant (Figure 9A, Table S7). Conversely, eight genes, *LOC_Os04g52810* (*OsONAC083*), *LOC_Os04g27190* (*OsTPS19*), *LOC_Os06g11200*, *LOC_Os05g06814, LOC_Os06g15990* (*OsALDH2B1*), *LOC_Os01g45720*, *LOC_Os05g17604* (*SHR5-RLK*), and *LOC_Os11g31470*, were consistently downregulated (Figure 9B, Table S7).

**Figure 10:**
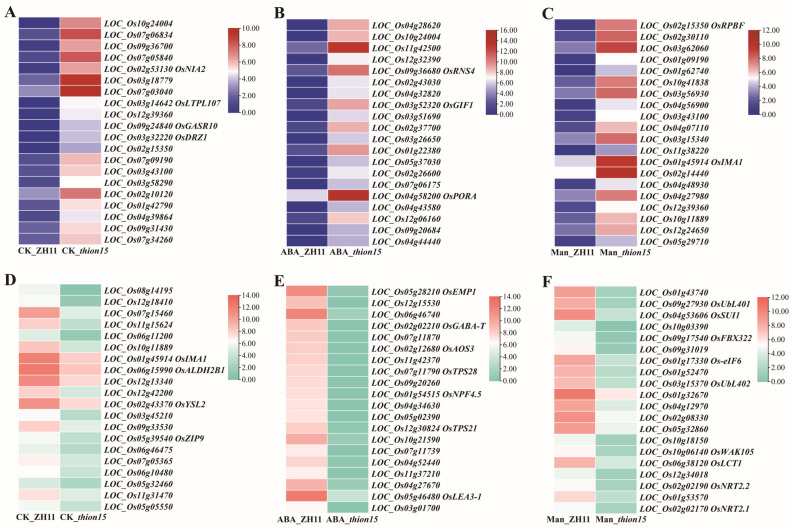
Heatmap representation of the expression profiles for the top 20 genes exhibiting the largest differential expression between *Osthion15* and ZH11. (**A**–**C**) The top 20 genes with the most significant upregulation in expression levels under CK (**A**), ABA stress (**B**), and drought stress (**C**). (**D**–**F**) The top 20 genes with the most significant downregulation in expression levels under CK (**D**), ABA stress (**E**), and drought stress (**F**).

Further analysis focused on the 20 most significantly upregulated and downregulated genes in *Osthion15* under the three conditions. Among the upregulated genes, several were associated with ABA and drought responses. Under normal conditions, *LOC_Os02g53130* (*OsNLA2*) and *LOC_Os03g32220* (*OsDRZ1*) were identified as ABA- and drought-related genes. Under ABA stress, gibberellin-related genes *LOC_Os09g24840* (*OsGASR10*) and *LOC_Os03g14642* (*OsLTPL107*), as well as ABA-responsive genes, such as *LOC_Os09g36680* (*OsRNS4*), and *LOC_Os03g52320* (*OsGIF1*), were upregulated. Additionally, the chlorophyll synthesis-related gene *LOC_Os04g58200* (*OsPORA*) exhibited increased expression. Under drought stress, transcription-activating factor *LOC_Os02g15350* (*OsRPBF*) and iron transport-related peptide *LOC_Os01g45914* (*OsIMA1*) were among the significantly upregulated genes (Figure 10A–C, Table S8).

Among the downregulated genes, those under normal conditions included metal transport-related genes (*LOC_Os12g18410*, *LOC_Os01g45914* (*OsIMA1*), *LOC_Os02g43370* (*OsYSL2*), and *LOC_Os05g39540* (*OsZIP9)*) and plant immune-related genes (*LOC_Os07g15460* (*OsNRAMP1*) and *LOC_Os06g15990* (*OsALDH2B1*)). Under ABA stress, the downregulated genes were primarily involved in ABA and drought responses, such as *LOC_Os05g28210* (*OsEMP1*), *LOC_Os02g02210* (*OsGABA-T*), and *LOC_Os05g46480* (*OsLEA3-1*), as well as pest-related genes *LOC_Os07g11790* (*OsTPS28*) and *LOC_Os12g30824* (*OsTPS21*). Under drought stress, ubiquitination-related genes (*LOC_Os09g27930* (*OsUbL402*), LOC_Os09g31019 (ubiquitin fusion protein), and *LOC_Os03g15370* (*OsUbL402*)), as well as drought-responsive gene LOC_Os04g53606 (*OsSUI1*), cell wall-associated receptor kinase *LOC_Os10g06140* (*OsWAK105*), and nitrate transporters *LOC_Os02g02170* (*OsNRT2.1*) and *LOC_Os02g02190* (*OsNRT2.2*), were significantly downregulated (Figure 10D–F, Table S8). These findings highlight the complexity and multifaceted nature of the molecular mechanisms underlying the sensitivity of *Osthion15* to drought and ABA stress. The differential expression of genes involved in diverse physiological processes indicates that *OsTHION15*, a small peptide, likely regulates rice stress responses through multiple pathways.

### 2.10. Expression Profiles of Stress-Responsive Genes

**Figure 11:**
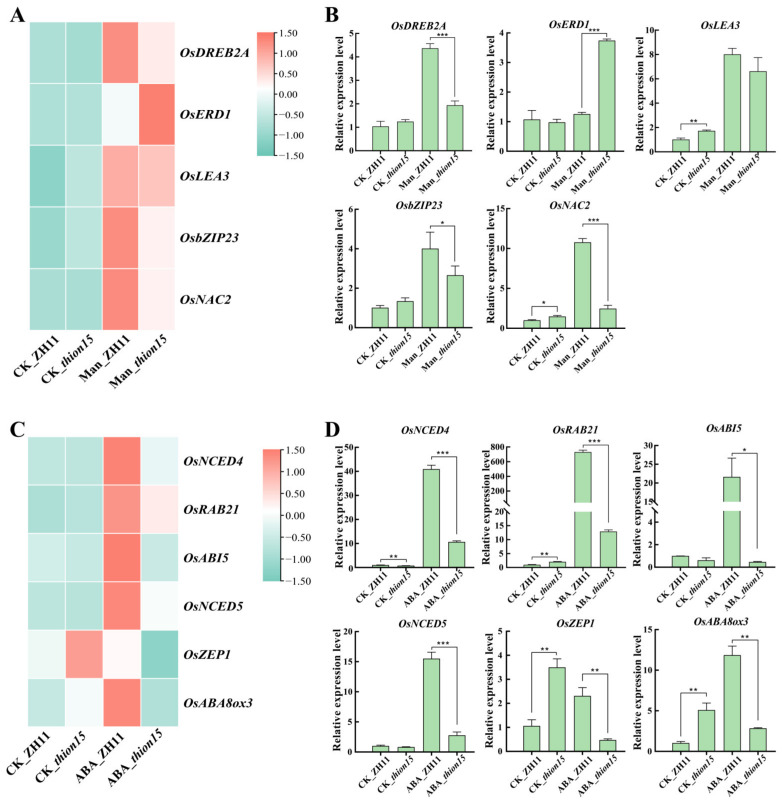
Expression pattern of stress-response genes under drought and ABA stress. (**A**) Expression heatmap of drought-stress-responsive genes. (**B**) qRT-PCR results of drought-stress-responsive genes. Data are mean values ± SDs. (**C**) Expression heatmap of ABA-stress-responsive genes. (**D**) qRT-PCR results of ABA-stress-responsive genes. Data are mean values ± SDs. *, *p* < 0.05, **, *p* < 0.01, ***, *p* < 0.001 by one-way ANOVA.

To further investigate the mechanisms underlying the heightened sensitivity of the *Osthion15* mutant to drought and ABA stress, we analyzed the expression profiles of stress-responsive genes. Under drought stress, five drought-responsive genes were significantly upregulated in the wild-type ZH11, whereas the *Osthion15* mutant exhibited distinct expression patterns. Notably, three key transcription factors, i.e., *OsDREB2A*, *OsBZIP23*, and *OsNAC2*, showed significantly lower expression in the *Osthion15* mutant compared to ZH11 (Figure 11A). qRT-PCR validation confirmed these findings, demonstrating that the *Osthion15* mutant exhibited reduced upregulation of *OsDREB2A*, *OsBZIP23*, and *OsNAC2* under drought stress (Figure 11B). These results suggest that the diminished activation of these transcription factors in the *Osthion15* mutant contributes to its increased sensitivity to drought conditions. Under ABA stress, six ABA-responsive genes were significantly upregulated in ZH11 (Figure 11C). However, in the *Osthion15* mutant, *OsNCED4*, *OsRAB21*, and *OsNCED5* were expressed at significantly higher levels than in ZH11, whereas *OsABI*, *OsZEP1*, and *OsABA8ox3* were downregulated (Figure 11C). qRT-PCR analysis further confirmed these differential responses, revealing significant variations in the expression of these six genes between the *Osthion15* mutant and ZH11 under ABA stress (Figure 11D). These findings indicate that while the *Osthion15* mutant exhibits an enhanced response to ABA stress for *OsNCED4*, *OsRAB21*, and *OsNCED5*, it shows a diminished or impaired lost response for *OsABI*, *OsZEP1*, and *OsABA8ox3*. Overall, these results demonstrate that *OsTHION15*, as a small peptide, plays a critical role in modulating gene expression under stress conditions. Its absence in the *Osthion15* mutant disrupts the regulation of key stress-responsive genes, leading to altered responses to drought and ABA stress. These results highlight the multifaceted role of *OsTHION15* in coordinating plant stress adaptation.

## 3. Discussion

The *OsTHION* gene family, characterized by cysteine-rich signal peptides, plays a pivotal role in plant stress responses, particularly in disease resistance and immunity. This study provides a comprehensive analysis of the OsTHION family in rice, focusing on its evolutionary relationships, expression patterns, and functional roles under various stress conditions. Our findings reveal that *OsTHION* genes are induced not only by abiotic stress adaptation, such as drought, salt, and cold stress, but also by biotic stress responses. The importance of *OsTHIONs* in enhancing plant resilience to environmental challenges aligns with previous studies that highlight the multifunctional roles of cysteine-rich peptides in stress adaptation [39].

Consistent with previous results, our phylogenetic analysis classified 44 OsTHION family members into 4 distinct groups (Figure 1) [40]. Subgroups III and IV contained more members, and a greater number of their genes were induced by stress (Figure 4A), suggesting their significant involvement in plant adaptability. The evolutionary divergence between OsTHIONs and their *Arabidopsis* counterparts (AtTHIONs) suggests that the THION family has undergone significant functional differentiation between monocot and dicot (Figure S1). This divergence likely reflects the adaptation of OsTHIONs to specific environmental and developmental challenges faced by rice, a staple crop often exposed to fluctuating stress conditions. The lack of collinearity between OsTHIONs and AtTHIONs further supports the hypothesis that these genes have evolved distinct regulatory and functional roles in different plant lineages (Figure S2), consistent with findings in other plant gene families [41]. The promoter regions of *OsTHION* genes are enriched with cis-acting elements associated with light response, hormone signaling, and stress responses (Figure 2). Notably, the abundance of ABRE (abscisic-acid-responsive elements) and CGTCA (jasmonic-acid-responsive elements) highlights the involvement of OsTHIONs in hormone-mediated stress signaling pathways. This aligns with the observed upregulation of several *OsTHION* genes under ABA and drought stress, suggesting that these genes play integral roles in the ABA-dependent stress response mechanism in rice [42]. The presence of G-box elements, which regulate light-induced gene expression, further indicates that OsTHIONs integrate environmental signals to fine-tune stress responses, a mechanism also observed in other stress-responsive gene families [43].

The comprehensive analysis of *OsTHION* gene expression patterns across various tissues and stress conditions provides valuable insights into their functional roles in rice growth, development, and stress adaptation. Tissue-specific and stage-specific expression profiles of *OsTHION* genes, as revealed through the heatmap and qRT-PCR analyses (Figure 3A–D), suggest that these genes participate in diverse biological processes. The significant upregulation of *OsTHION15* under drought, salt, and cold stress (Figure 4A,B) and its plasma membrane localization (Figure 5G,H) suggest that it may function in stress signal perception or transduction. Additionally, the enhanced GUS signals in the *pOsTHION15::GUS* transgenic plants under drought stress (Figure 5F) further confirm its responsiveness to stress conditions. Its expression in reproductive tissues, particularly in the stigma and style at the FM stage (Figure 5D,E), suggests its involvement in reproductive processes, potentially regulating stamen–pistil interactions and fertilization.

The functional characterization of *OsTHION15* through *Osthion15* mutant analysis provides compelling evidence for its critical role in drought stress tolerance and ABA signaling in rice. The *Osthion15* mutants exhibited heightened sensitivity to drought stress, as evidenced by increased wilting, mortality, and reduced fresh weight under mannitol treatment (Figure 6B,C). These findings align with previous studies demonstrating that cysteine-rich peptides play essential roles in plant stress responses by modulating cellular signaling and stress adaptation mechanisms [44,45]. The severe drought sensitivity of the *Osthion15* mutants underscores the importance of *OsTHION15* in maintaining cellular homeostasis and stress resilience under water-deficient conditions. The involvement of *OsTHION15* in the ABA signaling pathway further highlights its multifaceted role in stress adaptation. The presence of ABA-responsive cis-acting elements, such as ABREs, in the promoter region of *OsTHION15* (Figure S3) suggests that its expression is regulated by ABA, a key hormone mediating plant responses to drought and other abiotic stresses [46,47]. The pronounced growth defects observed in the *Osthion15* mutants under ABA stress indicate that *OsTHION15* is integral to ABA-mediated stress responses (Figure 7B,C).

The DEG analysis of *Osthion15* and ZH11 under drought and ABA stress provides critical insights into the molecular mechanisms regulated by *OsTHION15* in rice. The significant upregulation of *OsTHION15* under drought, salt, and cold stress (Figure 4) highlights its central role in abiotic stress adaptation. The RNA sequencing data reveal distinct transcriptional responses in the *Osthion15* mutants compared to ZH11, with 414 DEGs under normal conditions, 912 DEGs under drought stress, and 6660 DEGs under ABA stress (Figure 8A–C, Table S6). These findings emphasize the extensive regulatory influence of *OsTHION15*, particularly under stress conditions. The GO and KEGG pathway analyses further elucidated the functional implications of these DEGs. Under ABA stress, pathways such as ribosome biogenesis, PPAR signaling, and plant hormone signal transduction were significantly enriched (Figure S4). Similarly, under drought stress, pathways related to photosynthesis, nitrogen metabolism, and glyoxylate and dicarboxylate metabolism were enriched (Figure S4). These results suggest that *OsTHION15* regulates stress responses by modulating key metabolic and signaling pathways.

The expression profiles of specific DEGs provide further evidence for the role of *OsTHION15* in stress adaptation. NAC transcription factors, known for their role in stress responses, were notably affected in the *Osthion15* mutants. For instance, overexpression of the NAC gene OsNAC6/SNAC2 in rice has significantly enhanced tolerance to multiple abiotic stresses, including drought, high salinity, and cold, particularly during the seedling developmental stage [48,49]. *ONAC083* (*LOC_Os04g52810*) was downregulated under the control, drought, and ABA conditions in the *Osthion15* mutants compared to ZH11 (Figure 9B, Table S7). These findings suggest that *OsTHION15* may modulate the stress response through NAC transcription factors.

**Figure 12:**
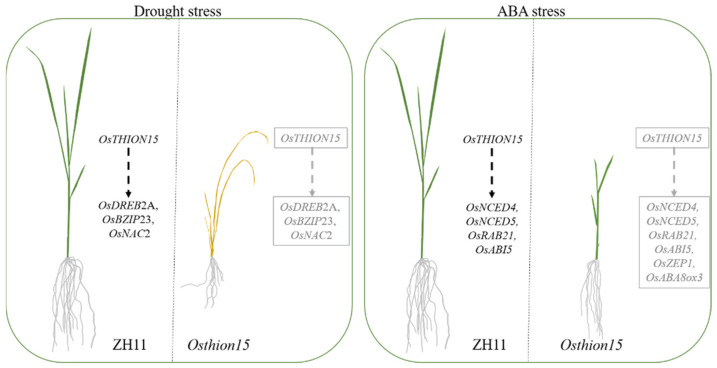
Model showing the role of *OsTHION15* under drought and ABA stress. (The dotted arrows indicate enhanced expression).

Compared to the wild-type ZH11, the *Osthion15* mutants exhibited significantly lower expression levels of key drought stress response genes (*OsDREB2A*, *OsBZIP23*, and *OsNAC2*) after drought stress (Figure 11A,B). Previous studies have shown that overexpression of these genes enhances drought tolerance in rice [50,51,52], suggesting that the reduced expression of these genes in *Osthion15* mutants contributes to impaired drought resistance. Under ABA treatment ABA-stress-responsive genes (*OsNCED4*, *OsNCED5*, *OsRAB21*, and *OsABI5*) were significantly upregulated in ZH11, but exhibited lower expression levels in the *Osthion15* mutants. As *NCED* genes encode key enzymes in ABA biosynthesis, their dysregulation in the *Osthion15* mutants suggests a disruption in ABA homeostasis, ultimately affecting plant stress responses [53]. This suggests that *OsTHION15* may influence ABA levels by regulating the expression of *OsNCED4* and *OsNCED5*, thereby participating in the plant stress response. *OsABI5*, a key transcription factor in the ABA signaling pathway [54], was downregulated in the *Osthion15* mutants, likely contributing to their increased sensitivity to ABA stress. Surprisingly, ABA-induced genes (*OsZEP1* and *OsABA8ox3*) were also significantly downregulated in the *Osthion15* mutants under ABA stress, further indicating that *OsTHION15* is essential for proper ABA-mediated stress adaptation. These results suggest that *OsTHION15* may regulate gene expression through multiple complex pathways, thereby playing a critical role in modulating plant stress responses (Figure 12). In conclusion, this study demonstrates that *OsTHION15* is a critical regulator of the drought response and ABA signaling in rice. Its loss of function increases sensitivity to drought and ABA stress, highlighting its importance in stress adaptation. These findings provide valuable insights into the molecular mechanisms underlying stress responses in rice and suggest that *OsTHION15* could be a potential target for genetic engineering to improve stress resilience in crops. The comprehensive analysis of OsTHION gene expression patterns across various tissues and stress conditions provides valuable insights into their functional roles in rice growth, development, and stress adaptation. While our study focused on cultivated rice (Zhonghua 11), wild rice species (*O. rufipogon*, *O. nivara*, etc.) represent a promising avenue for future research. Wild rice exhibits enhanced stress tolerance due to its broader genetic diversity and adaptive evolution under natural selection pressures [55]. The newly constructed Oryza super-pangenome (comprising 13 wild and cultivated species) revealed that only ~10% of gene families are conserved across the genus, while the remaining 90% represent untapped genetic diversity, including numerous stress-related gene clusters absent in domesticated rice [56]. Future studies could leverage wild rice genomic resources to identify superior OsTHION alleles for breeding applications.

## 4. Materials and Methods

### 4.1. Phylogenetic Analysis of OsTHION Gene Families

A total of 44 OsTHION protein sequences were retrieved from the Phytozome V13 database through the genome version of *Oryza sativa* (MSN v7.0, Phytozome genome ID: 323 and NCBI taxonomy ID: 39947) [57,58] (https://phytozome-next.jgi.doe.gov/) (accessed on 12 August 2024) (Table S1). Multiple sequence alignment of these OsTHIONs was performed using MUSCLE (https://www.ebi.ac.uk/Tools/msa/muscle/) (accessed on 12 August 2024). Phylogenetic reconstruction and molecular evolutionary analyses were conducted using MEGA 12 software (https://www.megasoftware.net) (accessed on 12 August 2024), employing the maximum likelihood method with 1000 bootstrap replicates [59]. Additionally, 62 members of the AtTHION family were identified through BLAST analysis of the 44 OsTHION protein sequences (Table S2). The same approach was used to construct the fusion evolutionary tree (Figure S1).

### 4.2. Analysis of Cis-Acting Regulatory Elements in OsTHION Gene Promoters

The 2.5 kb upstream sequence of the 44 *OsTHION* genes was extracted from Phytozome 13 (Table S1). *Cis*-acting regulatory elements associated with light responsiveness, phytohormone responsiveness, plant growth regulation, and stress responsiveness were predicted using the Plant *Cis*-Acting Regulatory Element (PlantCARE, http://bioinformatics.psb.ugent.be/webtools/plantcare/html/) (accessed on 16 August 2024) [60]. The distribution patterns of these regulatory elements were visualized using the R programming language (version 4.1.2).

### 4.3. Expression Profiling of OsTHIONs in Different Tissues and Under Stress Conditions

The FPKM (Fragments Per Kilobase of transcript per Million mapped reads) values of the *OsTHION* genes were obtained from the rice eFP browser [36]. Expression levels were normalized and transformed to log2 values, with pixel intensities corresponding to the relative expression levels. The tissue-specific expression heatmap was generated using TBtools software (Version 1.098) [61]. Plant tissue samples were collected from ZhongHua 11, including roots and leaves of 14-day seedlings, mature flower organs (inflorescence, stigma, and ovary), and four developmental stages ovules (AC, MMC, FM, and MO). Total RNA was extracted using an E.Z.N.A. Total RNA Kit II (Omega, Norcross, GA, USA), followed by cDNA synthesis with reverse transcriptase for subsequent qRT-PCR analysis. To validate *OsTHION* expression patterns under abiotic stresses, we performed qRT-PCR analysis using ZH11 seedlings. Fourteen-day-old seedlings grown in complete rice nutrient solution were subjected to three stress treatments (salt stress: 150 mM NaCl, drought stress: 200 mM mannitol, and cold stress: 4 °C) starting at 8 am. Complete seedling tissue samples were collected at 0, 1, 3, 6, 12, and 24 h post-treatment for total RNA extraction and subsequent qRT-PCR analysis. To validate the efficacy of the drought, salt, and cold stress treatments, we analyzed the expression of stress-responsive marker genes (drought stress genes: OsDREB2A and OsNAC2, salt stress genes: OsHAK9 [62] and OsHAK21 [63], cold stress genes: OsCBF1 [64] and OsTPP1 [65]). The results demonstrated that OsDREB2A and OsNAC2 were significantly upregulated under drought stress, OsHAK9 and OsHAK21 were strongly induced under salt stress, and OsCBF1 and OsTPP1 exhibited marked increases in expression under cold stress (Figure S6).

### 4.4. Histochemical GUS Assay

The *pOsTHION15::GUS* construct was generated by amplifying a 2.5 kb *Os*THION15 promoter fragment from ZH11 genomic DNA using gene-specific primers (Table S9). The amplified fragment was subsequently cloned into the pGWB533 vector through BP and LR recombination reactions. Transgenic rice plants were generated via *Agrobacterium tumefaciens*-mediated transformation of rice calli [66]. Histochemical GUS staining was performed by incubating fresh rice tissues overnight in a GUS staining buffer at 37 °C. The samples were subsequently decolorized in acetone and visualized using a Leica M205 FA stereomicroscope.

### 4.5. Subcellular Localization of OsTHION15

For subcellular localization analysis, the *35S::OsTHION15::GFP* fusion construct was generated. The coding sequence of *OsTHION15* was amplified from ZH11 cDNA using specific primers (Table S9) and cloned into the pGWB505 vector through BP and LR reactions. Both the *p35S::OsTHION15::GFP* and *p35S::GFP* (control) constructs were transiently expressed in *Nicotiana benthamiana* leaves via *Agrobacterium*-mediated transformation. The transfected leaves were maintained at 22 °C for 48 h, and GFP fluorescence was detected using a Leica SP8 confocal laser scanning microscope.

### 4.6. Osthion15 Mutant Obtained by CRISPR/Cas 9

Using CRISPR/Cas9 gene editing technology, the target sequence “CCGTTTTGCGCATGGCACAAGGG” of the *OsTHION15* gene was mutated precisely. Following rice genetic transformation, we obtained T1 generation *Osthion15* mutants. These transgenic plants were subsequently grown, segregated, and screened to eliminate those containing the CRISPR vector. Through PCR identification using *OsTHION15*-ID-F/R primers and subsequent Sanger sequencing analysis, we successfully isolated two homozygous mutant lines: *Osthion15-1* and *Osthion15-2*. Both mutants were confirmed to carry frameshift mutations that disrupt the normal coding sequence of the OsTHION15 protein.

### 4.7. Stress Treatment Assays

For osmotic stress treatment, the ZH11 wild-type and *Osthion15* mutants (*Osthion15-1* and *Osthion15-2*) were cultivated in 800× Yoshida rice nutrient solution (Coolaber, Beijing, China) for 15 days. They were then transferred to a nutrient solution supplemented with 150 mM mannitol. The control plants were maintained in standard Yoshida nutrient solution without mannitol supplementation. After 10 days of treatment, the fresh weight of both the treated and control plants was measured to assess the physiological response to osmotic stress.

For ABA response analysis, seedlings were grown in sterilized containers with 1/2 Murashige and Skoog (MS) medium with 2 µM ABA. The control plants were maintained on an ABA-free medium. All plants were grown under 16 h light/8 h dark photoperiod at 30 °C. Shoot and root lengths were measured after 20 days of growth.

### 4.8. RNA-Seq Data Analysis

RNA sequencing was performed on two-week-old ZH11 wild-type and *Osthion15-1* mutant plant seedlings treated with either 150 mM mannitol or 2 µM ABA for three days. Three biological replicates were analyzed for each condition. Library preparation and sequencing were constructed by Novogene using the Illumina platform. Differential expression analysis was performed, and the results were visualized using volcano plots and Venn diagrams. Raw RNA-seq data were first subjected to quality control using fastp to remove adapter sequences, low-quality bases, and short reads [67,68]. Clean reads were aligned to the reference rice genome using HISAT2. Differential gene expression analysis was performed using DESeq2 [68,69], and the results were visualized through volcano plots and Venn diagrams. Gene Ontology (GO) and Kyoto Encyclopedia of Genes and Genomes (KEGG) enrichment analyses of the differentially expressed genes were conducted using the clusterProfilerr R package [70].

### 4.9. RNA Extraction and Quantitative Real-Time PCR

Total RNA was isolated from the drought-treated, ABA-treated, and control seedlings using an RNA extraction kit (OMEGA, Georgia and USA). cDNA synthesis was performed using a PrimerScript™ RTase Kit (TAKARA, Tokyo and Japan). Quantitative PCR was carried out in a 20 μL reaction volume containing 10 μL of 2× TransStar Top Green qPCR SuperMix (TransGen, Peking and China), 1 μL of cDNA template, and 0.4 μL of gene-specific primers (Table S9). The thermal cycling conditions were 95 °C for 30 s, followed by 40 cycles of 94 °C for 5 s and 60 °C for 15 s. Relative gene expression levels were calculated using the 2^–ΔΔCt^ method.

## Data Availability

The original contributions presented in this study are included in this article/the Supplementary Materials. Further inquiries can be directed to the corresponding author(s).

## Supplementary Materials

The following supporting information can be downloaded at: https://www.mdpi.com/article/10.3390/ijms26073447/s1.
