# Supplementary material for: Genome-Wide Identification and Expression Analysis of Thionin Family in Rice (Oryza sativa) and Functional Characterization of OsTHION15 in Drought Stress and ABA Stress
Source: Int J Mol Sci. 2025 Apr 7;26(7):3447. doi: 10.3390/ijms26073447 (PMC11989618; doi:10.3390/ijms26073447)
Supplement: Supplementary file 1 [file ijms-26-03447-s001.zip › Supplementary File/Figure S1-S5.pdf]

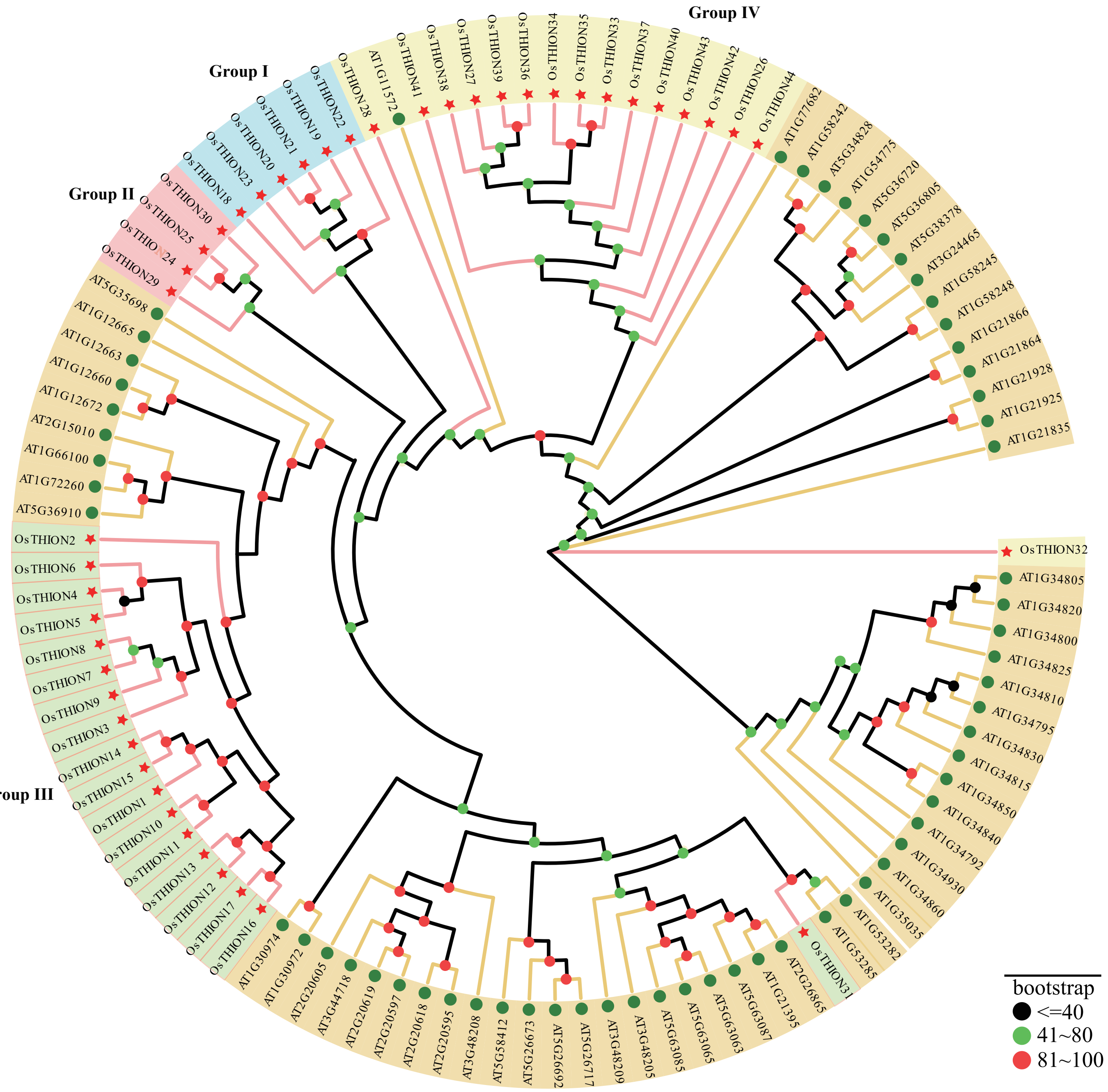

Figure S1. Mixed phylogenetic tree analysis of the coding sequences of THION family genes between rice and Arabidopsis.

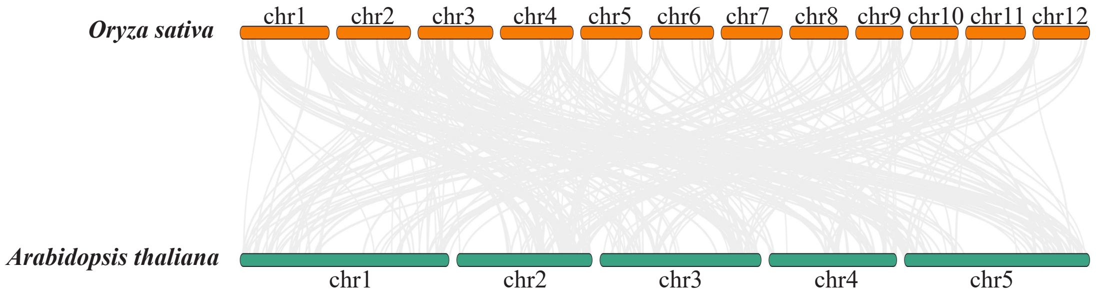

Figure S2. Collinearity analysis of between OsTHIONs and AtTHIONs

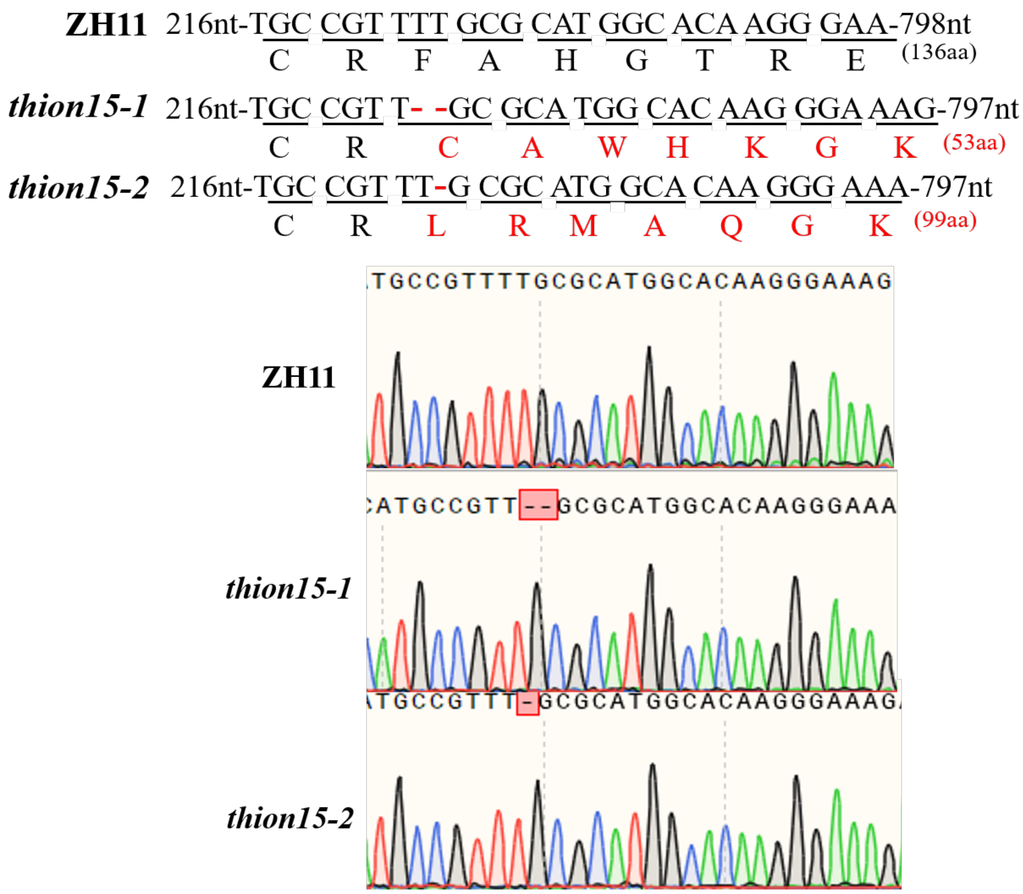

Figure S3. Gene structure and mutation sites of *OsTHION15* in *Osthion15* mutant lines. The base insertions or deletions, and the amino acid deletions or substitutions are indicated in red color.

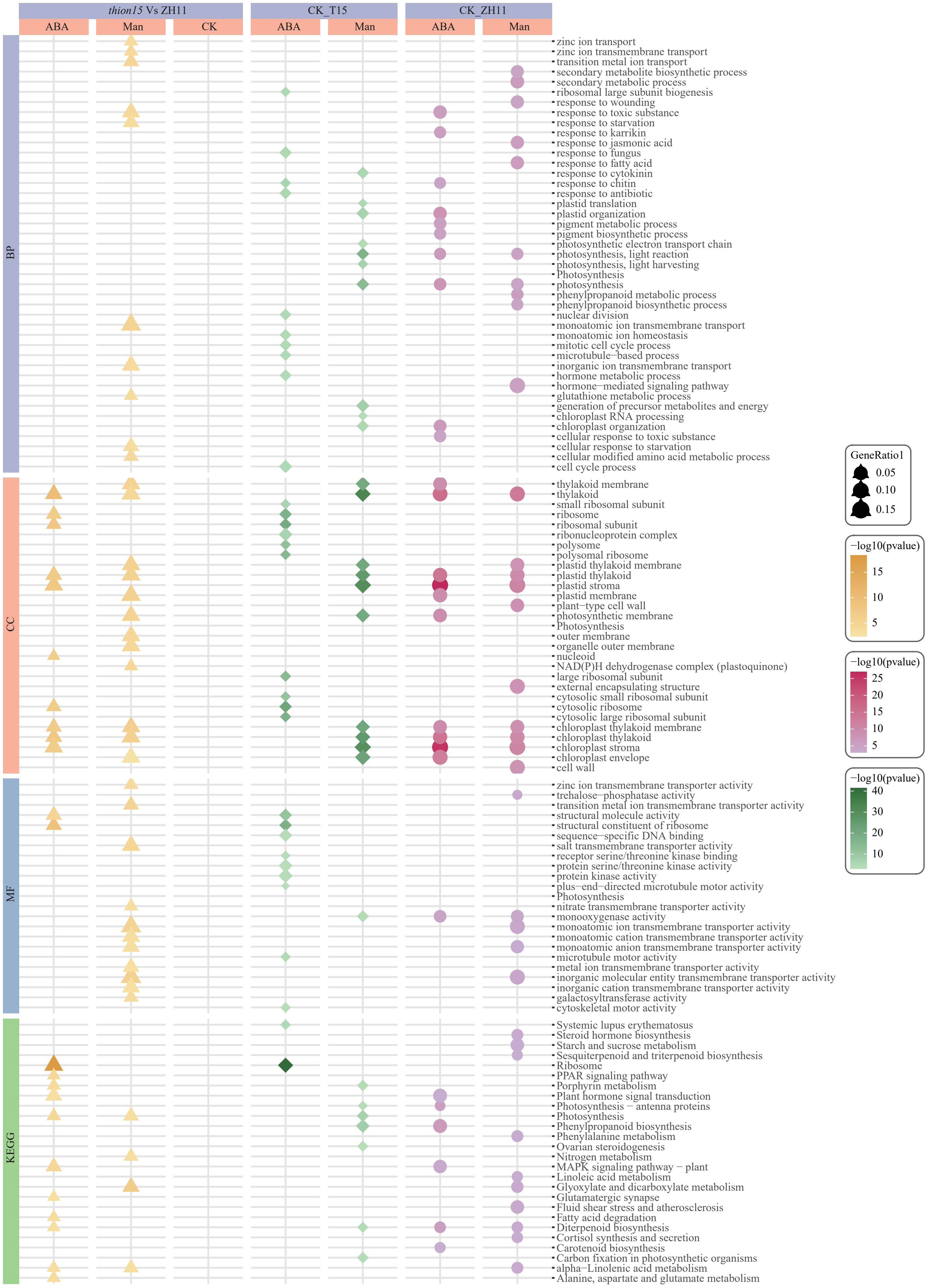

Figure S4. GO and KEGG pathway analyses of DEGs between *Osthion15* and ZH11 under CK, drought and ABA stress.

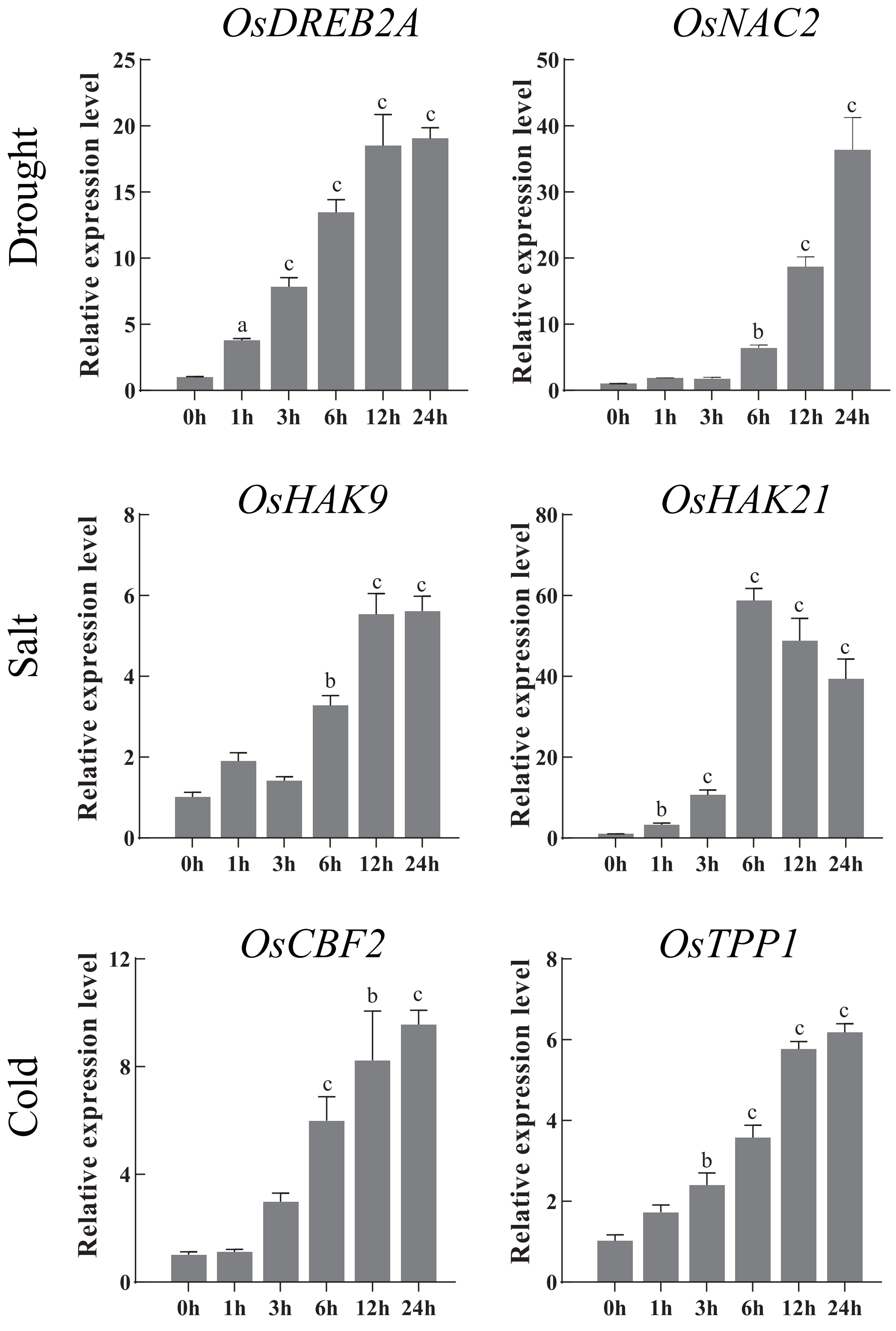

Figure S5. The expression validation of stress-responsive marker genes under stress treatments.
